# Adaptation and Feasibility Study of a Digital Health Program to Prevent Diabetes among Low-Income Patients: Results from a Partnership between a Digital Health Company and an Academic Research Team

**DOI:** 10.1155/2016/8472391

**Published:** 2016-10-27

**Authors:** Valy Fontil, Kelly McDermott, Lina Tieu, Christina Rios, Eliza Gibson, Cynthia Castro Sweet, Mike Payne, Courtney R. Lyles

**Affiliations:** ^1^Division of General Internal Medicine, University of California, San Francisco (UCSF) at Zuckerberg San Francisco General Hospital and Trauma Center, P.O. Box 1364, San Francisco, CA 94143, USA; ^2^Center for Vulnerable Populations, University of California, San Francisco (UCSF) at Zuckerberg San Francisco General Hospital and Trauma Center, P.O. Box 1364, San Francisco, CA 94143, USA; ^3^Omada Health, 500 Sansome St., Suite 200, San Francisco, CA 94111, USA

## Abstract

*Background*. The feasibility of digital health programs to prevent and manage diabetes in low-income patients has not been adequately explored.* Methods*. Researchers collaborated with a digital health company to adapt a diabetes prevention program for low-income prediabetes patients at a large safety net clinic. We conducted focus groups to assess patient perspectives, revised lessons for improved readability and cultural relevance to low-income and Hispanic patients, conducted a feasibility study of the adapted program in English and Spanish speaking cohorts, and implemented real-time adaptations to the program for commercial use and for a larger trial of in multiple safety net clinics.* Results*. The majority of focus group participants were receptive to the program. We modified the curriculum to a 5th-grade reading level and adapted content based on patient feedback. In the feasibility study, 54% of eligible contacted patients expressed interest in enrolling (*n* = 23). Although some participants' computer access and literacy made registration challenging, they were highly satisfied and engaged (80% logged in at least once/week).* Conclusions*. Underserved prediabetic patients displayed high engagement and satisfaction with a digital diabetes prevention program despite lower digital literacy skills. The collaboration between researchers and a digital health company enabled iterative improvements in technology implementation to address challenges in low-income populations.

## 1. Introduction

Nearly half of Americans will develop a chronic disease such as diabetes during their lifetime [1]. Optimal management of chronic diseases can be very complex and requires activating patients to proactively engage in self-management that includes behavioral changes and execution of complex medical treatment regimens needed to achieve optimal control of the disease. To this end, interventions that provide support for self-management have become a cornerstone for health system innovations toward preventing and treating chronic diseases such as diabetes [2–4]. The diabetes prevention program (DPP) was a landmark trial of an intensive lifestyle intervention that reduced risk for development of type 2 diabetes by 58% after 3 years. Subsequent practice-based interventions have validated the effectiveness of DPP in weight reduction and preventing diabetes in real-world clinical settings [5, 6]. As a result, the Center for Disease Control has established the National Diabetes Prevention Program to disseminate DPP programs across the country [7].

Advancements in information technology (IT) have expanded the ability to engage patients in the healthcare process, motivate health behavior change, and offer the potential to disseminate lifestyle self-management programs like DPP on a large scale [8]. Digital health tools (Internet- and/or mobile-phone-based) to enhance self-management of diabetes have proliferated rapidly [8, 9] with inconsistent but often positive results in increasing healthy lifestyle practices (e.g., diet and exercise) and affecting clinical outcomes like glycemic control [10, 11]. While there is great promise for these digital health interventions, our study focuses on two major gaps that need to be addressed in order to see more consistent and widespread effectiveness. First, digital health interventions for self-management need to utilize content and curricula that are based on validated evidence for behavioral change. Second, very few feasibility studies of digital health interventions have focused on real-world clinical settings that care for underserved populations at highest risk of developing diabetes and poor health outcomes associated with the disease [10, 12]. This is a very important gap in the literature because high risk populations with greater disease burden, such as older adults, low-income individuals, and ethnic minorities [13], are often the same groups associated with lower computer literacy and encounter greater challenges in accessing and using digital health technologies [14, 15].

The Omada Health Program® (formerly known as* Prevent*) is an Internet- and mobile-phone-based educational program modeled after the DPP lifestyle intervention, which includes small group support, personalized health coaching, a weekly curriculum, and digital tracking tools, including a wireless scale delivered to each participant's home. Participants are placed into a private online social network where they can discuss their progress toward their goals and provide each other with social support. Participants are encouraged to read and post weekly comments in this forum. The program starts with a 16-week intensive curriculum focusing on weight loss and continues with a 36-week curriculum focusing on weight maintenance. The online platform allows participants to asynchronously complete weekly lessons, privately message, text message, and call a health coach for individual counseling, track weight loss and physical activity using a wireless weight scale and pedometer, and monitor their engagement and weight loss progress. In a previous quasi-experimental pre- and postintervention study, the Omada Health Program was associated with significant reductions in body weight and A1C that were maintained after 2 years [16]. In 2014, Omada Health sought to adapt its program for vulnerable populations at high risk of diabetes. Healthcare institutions that serve predominantly low-income and uninsured patients in the USA are often referred to as the healthcare safety net [17, 18].

In this paper, we describe a collaboration between health services researchers at University of California, San Francisco and Omada Health to adapt the program through a real-world, user-centered process and test its feasibility in prediabetic patients at a large, urban, county-operated safety net clinic.

## 2. Methods

### 2.1. Objectives

The work began at Omada in 2014 with the goal of adapting the program for more vulnerable patient populations. This included a literacy and content overhaul of the existing program, the first (beta) version of a Spanish-language version, and then a usability test of the modified content within a real-world clinic setting. To this end, Omada partnered with researchers at the UCSF Center for Vulnerable Populations (CVP) at the Zuckerberg San Francisco General Hospital and Trauma Center (ZSFG) to collaborate on this process.

More specifically, the partnership between Omada and UCSF had two primary objectives: (1) adapting the literacy level and cultural relevance of the online program content for low-income, underserved populations, using both focus groups (phase 1) and in-depth editing of the entire weekly curriculum (phase 2); (2) testing the feasibility and acceptability of the modified program in a small sample of patients at a large safety net clinic (using observations of in-person registration and follow-up phone interviews, phase 3), with the overall goal of using the results from this work to inform further improvement of the program (phase 4).

### 2.2. Research Setting

From June to November 2015, we recruited patients receiving primary care at the Richard H. Fine People's Clinic, an adult primary care clinic based in ZSFG. This clinic serves 6,000 low-income patients in the city and county of San Francisco, among whom about three-fourths have public insurance, three-fourths are nonwhite, 40% prefer to speak a language other than English, and 15% are monolingual Spanish speakers. This same clinic was the study setting for all phases of this work.

### 2.3. Research Approach

We used a user-centered approach to design a prototype. User-centered design involves incorporating the perspectives and experiences of end-users in planning, designing, and finalizing a technology or tool with the ultimate goal of improving usability, acceptability, and value to potential users [19]. User-centered design is increasingly being used to inform the design of health technologies, including those geared toward facilitating lifestyle management or the self-management of chronic conditions, including diabetes [19–21]. We employed user-centered design in a 4-phase approach to inform and iterate the design of a prototype adaptation of the program for patients receiving medical care in a safety net healthcare setting.

In phase 1, we conducted focus groups to understand the needs and perspectives of potential end-users. In phase 2 based on this feedback, we adapted the program's sign-up process and online curriculum. In phase 3, we conducted a feasibility study to test the modified program with safety net patients. In the final phase, we provided and adopted recommendations for the next iteration that will inform a larger controlled trial in safety net clinics throughout the region. The Institutional Review Board of the University of California, San Francisco approved the study.

#### 2.3.1. Phase 1: Assessing Perspectives and Preferences for Lifestyle Management

In June 2015, we recruited English speaking and Spanish speaking participants to participate in two language-specific focus groups. We recruited participants by contacting diabetic and prediabetic patients who were enrolled in upcoming in-person Spanish and English diabetes education classes, which were held on a biweekly or monthly basis at ZSFG. All patients were referred to these classes by their primary care provider team, and all were eligible for recruitment to participate in phase 1 focus groups if they were fluent in English or Spanish and able to give written informed consent.

The purpose of the focus groups was to assess the overall acceptability of the program (i.e., initial reaction, attitude, and baseline receptiveness to health tips for behavioral change) and inform content modifications that would better align the program's curricular content with socioeconomic conditions and sociocultural preferences in this population. Participants discussed their overall perspectives on the use of technology for lifestyle management. After reviewing direct excerpts from the curriculum, participants provided feedback about the clarity and relevance of the content. To document participant feedback during this phase, we had two research analysts taking detailed notes with verbatim quotes to capture the discussion in these group sessions.

The authors used a team-based approach to conduct descriptive qualitative content analysis of focus group discussions, directly informed from the semistructured interview questions that captured perceptions of the acceptability of the online weight loss platform and feedback on specific excerpts from the program's curricular content. The three leads of the focus groups (LT, CR, and CRL) independently reviewed the written notes from the focus group sessions to achieve a consensus on the original list of codes, which were then reviewed by the entire research team. Any discrepancies were resolved by consensus.

#### 2.3.2. Phase 2: Adapting the Program Curriculum for Readability and Relevance

Applying the feedback we received from the focus groups, we created an adaptation of the existing curriculum to improve the readability and relevance of the content for a safety net population. To assess the readability of the existing curriculum and guide the adaptation of the content, we used the SMOG readability formula, an index used to determine the grade level required to understand a written passage [22]. In adapting the readability of the curriculum, we followed recommendations from the US National Institutes of Health to aim for 6th-grade reading level or below when developing easy-to-read health materials [23], making further simplifications to address the high prevalence of limitations in literacy, health literacy, and English proficiency among our clinic population. We modified the content of the program lessons to achieve SMOG readability indices of 5th-grade (on average) reading levels, while preserving core concepts of the original lessons. We also adapted the content to address lifestyle preferences and limitations reflected by participants in phase 1 focus groups.

Once a lower literacy version of the program content was completed in English, it was then translated into Spanish. A bilingual, bicultural native Spanish speaker completed the Spanish translations. A second Spanish speaker reviewed and revised the final versions to ensure that the translated language was relevant to Latino patients and not just a direct literal translation of the English text. Because this was the first Spanish version of the program, we considered it a beta version with which to gain very early feedback about the content, rather than a complete adaptation into Spanish.

#### 2.3.3. Phase 3: Assessing the Feasibility of the Modified Program in a Safety Net Population

Next, our team moved from content adaptation to testing of the implementation of the program within a clinical setting. From August to November 2015, we recruited English speaking and Spanish speaking patients to participate in the phase 3 feasibility study, which followed two small prospective cohorts of English and Spanish speakers enrolling into the program at the same time. This feasibility study covered enrollment through the first 4 weeks of the core program.

We designed the recruitment protocol to be consistent with existing workflows for panel management in the clinic. To this end, we queried the electronic health record to identify patients who met eligibility criteria for language, age, Hemoglobin A1c (HbA1c) test result, and body mass index (BMI). We then sent the list to primary care providers to screen out individuals who were not suitable for the study (based on exclusion criteria) and refer additional patients that our electronic query may have missed. We also posted flyers at the clinic to allow individuals to self-refer (i.e., volunteer) for the study. Research staff called individuals who were referred by their providers or self-referred in response to posted flyers, verified eligibility via chart review and telephone screening, explained the purpose and procedures of the study, and scheduled in-person sessions to obtain written informed consent for phase 3 of the study.

Participants were eligible for phase 3 if they met all of the following eligibility criteria: (1) being fluent in English or Spanish; (2) age 18–75 at screening; (3) having had an HbA1c test result of 5.7–6.4% or fasting glucose test of 110–125 mg/dL in the past 6 months or as their most recent result; (4) BMI ≥ 24 kg/m^2^ (or ≥22 kg/m^2^ if Asian American); (5) using the Internet at least weekly; and (6) being able to give informed consent. We excluded participants who were already diagnosed with diabetes, taking any hypoglycemic medications, and had serious unmanaged mental health conditions (e.g., untreated bipolar disorder, severe untreated depression) or any other conditions that would preclude or make it difficult to participate in physical activity involving walking (e.g., severe arthritis and limited lower limb mobility). 

*Sign-Up Process*. We asked individuals interested in participating to attend an orientation session with a member of the study staff. During this session, participants received instructions adapted for low literacy on how to complete the sign-up process for the program. For participants of the English speaking cohort who expressed moderate to advanced comfort with computers, we asked participants to complete the sign-up process on their own time. For those with limited computer literacy, we allowed participants to complete the sign-up process during the orientation session, providing one-on-one assistance if needed. Since the Spanish version of the program was still in beta form, we asked all participants in the Spanish speaking cohort to complete the sign-up process in person to offer assistance if needed. We recorded the types of barriers experienced by participants who completed the sign-up process in person. Participants who successfully enrolled were placed in a small cohort with a common program start date. 

*Follow-Up Interviews*. Two and four weeks after the cohorts started the full 16-week program, we conducted semistructured phone interviews with study participants to explore barriers and facilitators to feasibility. At the two-week follow-up, we called all participants who had attended an orientation session. Interviews focused on (1) experiences of participants in completing the sign-up process, (2) getting oriented to the program, (3) using the online platform, (4) communicating with the health coach, (5) engaging with cohort members online, and (6) other barriers or facilitators to participating in the program. At the four-week follow-up, we called all participants who had been placed into a program cohort. Interviews focused on overall satisfaction and components of ongoing engagement with the program, including communicating with cohort members and the health coach, tracking changes in diet and exercise, and the feasibility and desire to apply new health knowledge and sustain engagement with the program.

#### 2.3.4. Phase 4: Applying Recommendations to Finalize a Prototype Program

Based on observations and perspectives from participants in signing up, getting oriented to, and engaging with the program, we provided recommendations for modifications to iterate the program in preparation for a controlled clinical trial in safety net clinics and for commercial deployments with safety net providers. We outlined overall barriers and potential strategies to address these challenges and recommended modifications to the program where feasible.

## 3. Results

### 3.1. Phase 1: Assessing Perspectives and Preferences for Lifestyle Management

We enrolled four English speaking and six Spanish speaking participants in phase 1 focus groups. Among the English speaking group, three participants were male, three had been diagnosed with diabetes, and one had been diagnosed with prediabetes. Among the Spanish speaking group, three were male and all six had been diagnosed with diabetes.

The majority of informants in both English and Spanish speaking focus groups were very receptive to the program's educational content, expressed a high level of interest in participating, and conveyed a willingness to change their behavior as a result of the program.

*I'm not just interested, I'm fascinated.*

*I need to wisen up a bit and stop being silly. These [health] tips would be helpful.*

However, our focus group discussions highlighted two limitations to the program's educational lessons that informed adaptation of the program to underserved populations who tend to receive care in safety net clinics. First, a few informants reported that the educational content was too complex. Participants suggested modifying the content to explain concepts that were difficult to understand in simpler terms.

*The word [placebo] looks familiar, but I haven't heard this before.*

*How do I calculate a serving? How can we calculate those daily percentages?*

Second, more than half of focus group informants suggested that the health tips contained in the lessons needed to be more practical. Many of the health tips did not resonate with participants because they did not align with the socioeconomic and sociocultural realities or preferences in this low-income population. For example, health tips on physical exercise requiring gym equipment were often impractical in this setting because access to fitness centers was limited. One participant noted the following:

*Affordability [of the gym] is something I find frustrating.*

In addition, the majority of informants expressed that finding motivation to exercise was often a challenge. Rather than more intense exercise regimens or going to the gym, participants expressed interest in walking or activities involving music as a motivator, such as dancing.

*Exercise can be about pleasure and not obligation.*

The Spanish speaking focus group additionally emphasized the importance of nutrition labels and false health claims. Participants had many questions about food labels such as “whole grain,” “organic,” and genetically modified organisms (GMOs). They suggested a need for Spanish language nutrition labels or having a glossary of simplified definitions for nutritional terms included in nutrition labels (e.g., sodium = salt).

*It's important to explain to people what are carbohydrates and other nutrients on the food labels. [Give] tips to know how to select foods that are whole grain.*

### 3.2. Phase 2: Adapting the Program Curriculum for Readability and Relevance

Changes made to the program curriculum to befit the literacy and cultural preferences of patients are shown in Table 1.

Specifically, to address concerns about the complexity of the curriculum, we adapted the readability level of each lesson (originally 9th grade or higher) to mostly a 5th-grade level or below. In addition to simplifying overall language, we simplified explanations of scientific concepts, preserving core concepts while improving understandability. To address the feasibility of and preferences for lifestyle changes, we adapted curriculum examples to fit perspectives expressed by phase 1 participants. In particular, we emphasized sources of motivation and exercises that did not require significant financial investments. For example, we replaced activities with potential financial burden (e.g., gym memberships, yoga classes, and races) with no or low-cost options (e.g., dancing, sports, and classes at community recreation centers). See Table 1 for examples of specific content changes. Additionally, to support healthy food choices that are accessible and culturally relevant, we added low-budget and ethnic recipe suggestions.

### 3.3. Phase 3: Assessing the Feasibility of the Modified Program in a Safety Net Population

**Figure 1:**
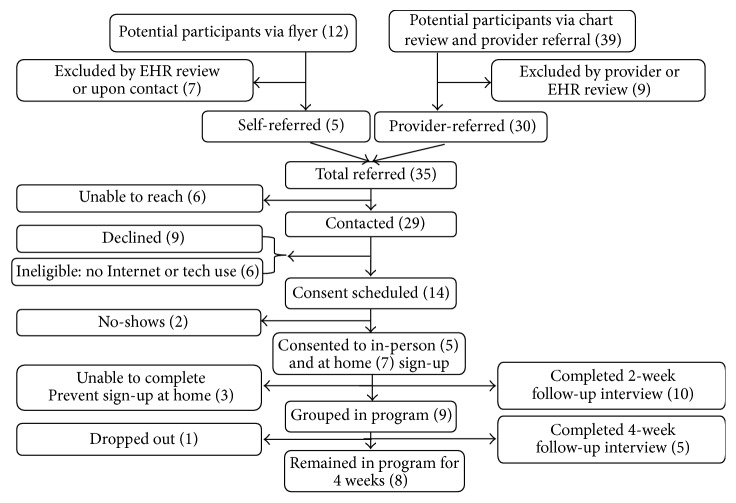
Flowchart of recruitment for phase 3 English-based feasibility study.

**Figure 2:**
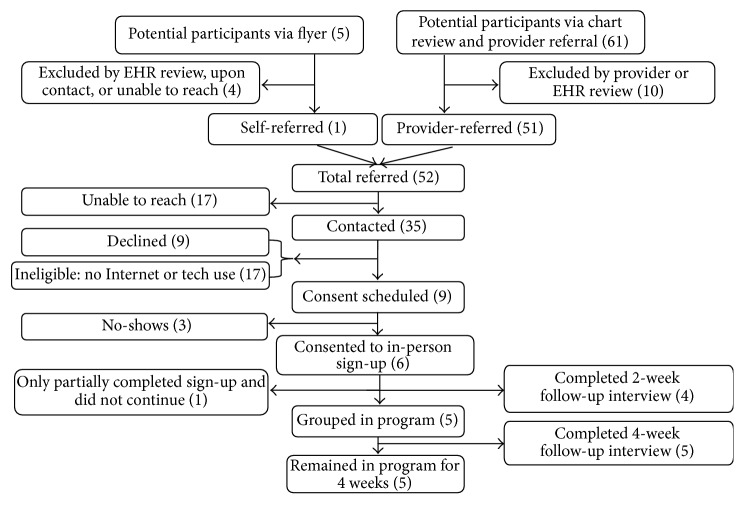
Flowchart of recruitment for phase 3 Spanish-based feasibility study.

During the feasibility study, we contacted 64 potentially eligible patients: 29 English speakers and 35 Spanish speakers [Figures 1 and 2]. A total of 6 English speakers (21%) and 17 Spanish speakers (49%) were not eligible because they did not use the Internet regularly. Another 9 English speakers (31%) and 9 Spanish speakers (26%) declined to participate, citing reasons of disinterest in research, current participation in another health program, or other personal or health issues.

Overall, 23 out of 41 eligible patients (54%) expressed interest in the program, and 18 of these eligible participants began the enrollment process (12 English speakers and 6 Spanish speakers). Ten of the 12 (83%) English speaking participants completed the two-week follow-up interview, and five of the nine actively enrolled in the program (55%) completed the four-week follow-up interview. Four of the six (67%) Spanish-speaking participants completed the 2-week follow-up interview, and all five (100%) of the actively enrolled Spanish speakers completed the 4-week follow-up interview.

#### 3.3.1. Sign-Up

The final feasibility sample was evenly split by gender (53% female), and the mean age was 53. Of the 12 participants of the English speaking cohort, 5 participants (42%) noted that English was not their first language.

All six Spanish speaking participants completed the sign-up process with guidance from a member of the research team. Seven English speaking participants wanted to complete the online sign-up process on their own at home; we asked five participants to complete the process in person due to limited experience/confidence using computers or the Internet. Of those 7 participants who were asked to complete sign-up independently at home, 4 (57%) finished the enrollment process. Among those who did not complete the sign-up process, it was mostly due to technical issues with their computer or Internet not working. One participant even stated,* “Just real guidance (would have helped me sign up)…I just got really frustrated.”*

Even among those who completed the sign-up process with one-on-one assistance, we observed several computer literacy challenges for a majority of participants: (1) many participants had difficulties using uniform resource locators (URLs). For example, some participants did not know where to type in the URL, while others did not know to press “enter” after typing in the URL. Participants made frequent errors (i.e., typos) in entering the URL or entered the URL into search bars leading to Google search results instead of access to the Omada website. This left participants confused and bewildered as they often could not recognize and correct their mistakes. (2) Navigating the online sign-up form was also challenging for some participants because they did not understand skip patterns that required users to click on an icon in order to move on to the next screen. (3) Participants also experienced challenges with the parts of the sign-up process that required using email (e.g., confirmation codes and links). Although they reported frequent Internet use, many participants rarely checked email and some participants did not have email accounts, requiring help to set up new ones.

#### 3.3.2. Engagement with the Weekly Lessons

*Weekly Logins*. There was a high level of engagement among feasibility study participants, with 80% signing in at least twice at the two-week follow-up, and an average of 4.7 logins per week for English speakers and 5.5 logins per week for Spanish speakers at the four-week follow-up. Of the program activities, participants engaged most frequently in group discussions, weigh-ins, and weekly lesson completion.

However, while logins overall were high, a few participants expressed technology accessibility barriers that mirrored their challenges in completing the sign-up process, such as not being able to remember their passwords to get back into the online content and having inconsistent access to the computer.

*Perceptions of the Curricular Content*. The majority of program participants who completed either the two-week or four-week interview reported satisfaction with the program and intentions to keep up with the healthy behaviors. Overall, participants also expressed that they thought the lessons were clear and useful.

*It's a lot of information that I never [heard] about. It's great for me, I tell my family, you have to go and read those lessons.*

*Balance, control, and food portions—that is the part that has helped me.*

Aside from a few participants who noted a lack of time to make lifestyle changes, participants noted that it was not difficult to complete the program's tracking requirements via food diaries and exercise/pedometer logs.

*I log my food every day… This program helps you have awareness of what you're doing.*

*Support from the Health Coach and Online Peer Group*. Among those who successfully completed the sign-up process and participated in a follow-up interview, all but one participant engaged with a health coach by telephone and 50% contacted the health coach by text message or email, in addition to telephone. The health coach at Omada reported that these participants were more interested in texting than other Omada users that were not part of this study. Participants stated clearly that they were the most satisfied with the direct support provided by the health coach throughout the program.

*She's very nice… If I have a question, she tells me the answer. I like talking to her.*

In general, about half of participants engaged in the online social network, though a few participants reported limited engagement with participating in online discussions with other peer cohort members, attributed to a lack of connection to cohort members or concerns about written literacy skills. Several participants expressed lower confidence with English proficiency and literacy when posting on their own.

*I'm not feeling any connection with the people that are in there. There's no camaraderie in seeing names in a chatroom.*

*I don't feel comfortable doing it on the computer. I'm not the writer. I don't spell it right, my sentences… I have to get my dictionary out.*

### 3.4. Phase 4: Recommendations for the Next Iteration

Finally, we made several modifications to the procedures, as outlined in Table 2. These changes provided additional guidance and assistance to address technical difficulties with signing up for and logging in to the program online, increase direct communication with the health coach, and facilitate stronger relationships between cohort members and other workflow process improvements. For example, we made a few modifications to the existing sign-up and orientation procedures for enrollment in the program, such as (1) creating step-by-step screenshot instructions for registration that all participants could take home with them, (2) creating video-based tutorials for interacting with the various features of the digital program, (3) creating and sending an additional email to participants letting them know more details about their upcoming group start dates (to reduce confusion and increase engagement before the program even began), and (4) providing the support staff phone number in more places during the sign-up process to ensure that participants could more easily access phone support at any step of the process.

## 4. Discussion

In this study, product developers from Omada Health partnered with health services researchers to utilize a user-centered approach to first adapt the online content of an existing digital health diabetes prevention program and then test the usability and feasibility of the modified product in a real-world safety net clinic that cares for underserved populations (e.g., low-income people, African Americans, and Latinos) at high risk of diabetes. While preserving the core elements of the original program, the adapted content was more aligned with the needs of the safety net patient population, written at a lower reading level, and incorporated tips that were more resonant with the preferences and sociocultural realities of the target population. Patients enrolled in the program remained engaged with high rates of regular use and reported overall satisfaction despite some substantial technical difficulties in signing up and logging into the program. This user-centered approach for feasibility testing is significantly underreported in the scientific literature, and we have provided detailed findings about how to design this approach to collect rich data within a short timeframe.

The diabetes prevention program (DPP) has been shown to prevent type 2 diabetes through lifestyle modification. Numerous studies have translated DPP to real-world community settings (e.g., YMCA, churches, or workplace) and clinical settings that required cultural adaptation of the program [24, 25]. To our knowledge, this is the first digital translation of DPP for an underserved patient population. A recent review found that among the 15 DPP translation studies, only one was conducted in a primary care clinical setting [24]. Our study makes important additions to the body of literature on translation of DPP in the real-world by providing specific recommendations about how the recruitment and initial enrollment process might be better completed within safety net clinical sites.

Challenges related to computer and online literacy and accessibility in signing up and logging into the program posed the biggest barriers for usability in this low-income population. Despite limiting our study to patients who reported weekly use of the Internet, a substantial portion of patients needed technical assistance to sign up for the program. In fact, even among those who we deemed more technically proficient and then attempted to sign up independently, more than one-third dropped out of the study because they could not complete the sign-up process due to technical difficulties related to poor computer or online literacy and inconsistent computer or Internet access. The finding of technical difficulties as a barrier to feasibility is not surprising. A recent systematic review of the impact of health IT on patient behavior change reported technical issues as a common barrier to usability of IT platforms [8].

Our study has uncovered a real opportunity for companies to collaborate with health services researchers embedded in safety net clinics to adapt and improve usability of digital health products in low-income and underserved populations. This partnership conferred several advantages that are rarely available in the field of digital health. First, the collaboration with a research center directly integrated with a safety net clinical practice allowed us to recruit a diverse sample of patients to test the product in a real-world process that was not separate from ongoing care for complex patients. Second, having the digital health company actively engaged as a partner on this work enabled our team to explore technical assistance solutions to improve usability of the technology in real-time (e.g., implementing step-by-step sign-up instructions within one week of identifying challenges in this process).

There are some limitations to consider in interpreting our findings. We designed this study as a feasibility study in a small sample of patients. Therefore, these findings are not necessarily generalizable to all high risk patients who receive care in safety net clinics. It is also possible that this safety net clinic provides care to a patient population associated with lower computer literacy than other safety net clinics. However, our user-centered process to iteratively adapt and test the Omada Health Program in a uniquely challenging patient population offers a potentially distinct advantage of refining an intervention toward maximal usability and sustainability. Because the clinic is a university-affiliated clinic located in San Francisco, its patients may be more receptive to clinical trials as well as technology solutions. This could have contributed to the level of interest in participating in the program and the sustained engagement among those who enrolled.

## 5. Conclusion

We documented patient interest, engagement, and satisfaction with a digital health diabetes prevention program among both English and Spanish speaking patients at a large safety net clinic. However, low computer and online literacy for some of this population presented implementation challenges that should be considered in digital health adaptations for low-income populations. The model of collaboration between researchers and a digital health company allowed for substantial iterations in the final program that can be scalable, improve usability, and contribute to increasing the overall impact of the product.

## Figures and Tables

**Table 1:** Examples of content and language modification to adapt online lessons in the Omada program to fit literacy levels and cultural relevance associated with low-SES patients in a large safety net clinic.

Original content		Adapted content		Adaptation
Quote	Readability index^*∗*^	Quote	Readability index^*∗*^	
*Proteins are the building blocks of muscle and tissue, making them essential to growth and healing. They support immune function, and are used to make hormones and enzymes. Protein is also a backup source of energy when carbohydrates aren't available. When it comes to eating protein, lean sources are great for your health*	6.0	*Protein builds muscles and tissue, and helps you grow and heal. Your body also uses protein as fuel when it runs out of carbs. Low-fat proteins are great for you*	1.8	Improve understandability by using fewer scientific terms

*Listening to music while you walk, waiting to read a favorite magazine or watch a guilty-pleasure TV show until you're on the stationary bike, or inviting your funniest friend to join you can turn exercise into something to look forward to *	18.2	*Listen to your favorite music while you walk, or invite a friend to join you. This can make exercise something to look forward to*	7.2	Emphasize exercises that do not require significant financial investments

*“Finally, pay attention to the circumstances and environments that inspire you to stay healthy, and soak up as much of that inspiration as you can. Playing sports, perfecting a new yoga pose, spending time in nature, being around kids, reading about people who overcome illness against all odds... Whatever inspires you, embrace that activity and plan to do it more often*	10.6	*Find the things that inspire you to stay healthy and use them to keep you on track. Maybe it's playing sports or being around kids. Maybe you enjoy spending time in nature or learning a new dance. Find what drives you, use it to stay inspired, and plan to do it more often*	1.8	Emphasize sources of motivation and activities that are accessible regardless of income level

*There's one more impressive stat about the DPP that you should know: 75% of the people who participated in the lifestyle program and met their first weight-loss goal, kept that weight off for three years. In comparison, only 5% of people who lose weight on traditional diets do the same*	9.2	*One more great thing about the program: people didn't just lose weight, but they kept the weight off. 3 out of 4 people kept the weight off for 3 years. With a regular diet, only 1 out of 20 people are able to keep the weight off*	3.8	Improve understandability by simplifying numerical concepts

^*∗*^While the entire lessons were on average at 5th-grade reading level, a few excerpts within a lesson could be at a higher SMOG index for readability based on the length of a sentence or the number of 3-syllable words.

**Table 2:** Program modifications to improve usability based on end-user feedback.

Observed challenges	Actions/strategies to address challenges
Lower computer literacy skills overall	Creating technical assistance tools for various stages of the program. Greater promotion of mobile phone interface and use of app for English speakers

Need for additional guidance to complete the sign-up process independently	Greater promotion of using Omada support staff for assistance. Creating technical assistance tools, that is, video tutorial, handouts with screenshots, and instructions for accessing the sign-up website and completing the account setup process

Need for additional participant communication about each step in the sign-up process	Modifying notification schedule to remind participants more often about where they were in the sign-up process and the expected launch date of the group cohort

Difficulty logging in to the program after signing up	More outreach from the program staff to help participants at the early stage

Desire for more connection with peers in the cohort	Offering a conference call session at the beginning of the program to get group members oriented and connected to one another offline
